# Serum Brain-Derived Neurotrophic Factor, and Plasma Catecholamine Metabolites in People with Major Depression: Preliminary Cross-Sectional Study

**DOI:** 10.3389/fpsyt.2018.00052

**Published:** 2018-02-28

**Authors:** Reiji Yoshimura, Taro Kishi, Kiyokazu Atake, Asuka Katsuki, Nakao Iwata

**Affiliations:** ^1^Department of Psychiatry, School of Medicine, University of Occupational and Environmental Health, Kitakyushu, Japan; ^2^Department of Psychiatry, School of Medicine, Fujita Health University, Toyoake, Japan

**Keywords:** 3-methoxy-4-hydroxyphenylglycol, homovanillic acid, brain-derived neurotrophic factor, major depression, serum, plasma

## Abstract

**Background:**

There are complicated interactions between catecholaminergic neurons and brain-derived neurotrophic factor (BDNF) in the brain. However, no reports have addressed the relationship among 3-methoxy-4-hydroxyphenylglycol (MHPG), homovanillic acid (HVA), and BDNF in the blood.

**Objective:**

This paper sought to investigate correlations between serum BDNF and plasma levels of MHPG and HVA in people with major depression (MD).

**Materials and methods:**

A total of 148 patients (male/female 65/83, age 49.5 ± 12.1 years old) who satisfied criteria for MD based on the Diagnostic and Statistical Manual of Mental Disorders IV were enrolled in the present study. Plasma levels of MHPG and HVA were analyzed using high-performance liquid chromatography, and serum BDNF was measured using ELISA.

**Results:**

No interactions were observed between plasma HVA levels (mean ± SD = 4.5 ± 1.5 ng/mL) and age, sex, HAMD scores, or serum BDNF levels (mean ± SD = 9.8 ± 2.9 ng/mL). No correlations were not also observed between plasma MHPG levels (mean ± SD = 5.9 ± 2.1 ng/mL) and age, sex, the HAMD17 scores (mean ± SD = 22.2 ± 2.9 ng/mL), or serum BDNF levels. Serum BDNF levels were negatively associated with HAMD17 scores.

**Conclusion:**

The results suggest that there are no significant correlations between catecholamine metabolites and BDNF in the blood for MDD patients.

## Introduction

Major depression (MD), which is the most prevalent and disabling form of depression, affects more than 30 million Europeans per year. In the USA, the estimated lifetime prevalence of MD is 16%. In addition to its disease burden, MD negatively impacts physical health (1). It has been speculated that catecholamines, neurotransmitters, norepinephrine, and/or dopamine play roles in MD (2), although the actions of catecholamines in the pathophysiology of MD remain incompletely understood. Norepinephrine exerts its effects by binding to α1- and β-adrenergic receptors, resulting in a stimulatory effect on cell signaling; in particular, such binding has been shown to increase intracellular phospholipase C and cyclic adenosine monophosphate (cAMP), respectively, whereas activation of the α_2_-adrenergic receptor suppresses intracellular cAMP and has an inhibitory influence on signaling (3). Drugs that inhibit the serotonin transporter (5-HTT) and/or the noradrenaline transporter exhibit anti-depressive efficacies. Selective serotonin reuptake inhibitors (SSRIs) and serotonin norepinephrine reuptake inhibitors (SNRIs) are first-line treatments for patients with MDD. We have demonstrated that milnacipran, an SNRI, increased plasma levels of 3-methoxy-4-hydroxyphenylglycol (MHPG), a major metabolite of norepinephrine, in the people with MD; this increase was related to milnacipran-associated clinical improvement in such patients (4). We have also reported that duloxetine, another SNRI, significantly increased plasma MHPG levels in the people with MD (5). Furthermore, in a series of prior studies, we demonstrated that the response to antidepressants was associated with plasma levels of catecholamine metabolites (6). Specifically, plasma levels of catecholamine metabolites, such as MHPG and homovanillic acid (HVA), could be used to predict response to SSRIs and SNRIs (4). The people with MD and lower plasma levels of MHPG had better response to milnacipran, whereas the people with MD and higher plasma levels of MHPG had better response to paroxetine. Given these findings, an increase in MHPG levels may play an important role in improving depressive symptoms. Furthermore, Yoon et al. (7) reported that for the people with MD, antidepressants decreased MHPG levels in cerebrospinal fluid (CSF) (7). Electroconvulsive therapy increased CSF HVA levels in the people with MD (8). In various ways, MHPG and HVA in plasma or CSF partially reflect brain status (4–8). Brain-derived neurotrophic factor (BDNF) is a neurotrophic factor that is abundant in the brain (9). BDNF is associated with neuroplasticity in the brain and plays a role in the pathophysiology of MD. According to a meta-analysis, blood levels (serum and plasma levels) of BDNF are lower in MDD patients than in healthy subjects. BDNF is synthesized and secreted in the brain, penetrates the blood–brain barrier, and is stored in platelets. The main source of BDNF in the blood is platelets, and BDNF is secreted in a calcium-dependent manner (10, 11). There exist interactions between catecholaminergic neurons and BDNF synthesis and secretion (12). We have reported that psychological stress is positively correlated with plasma MHPG and serum BDNF in healthy controls in workplaces in Japan (13, 14). There are complicated interactions between catecholaminergic neurons and BDNF in the brain. However, no studies have addressed the relationship among MHPG, HVA, and BDNF in the blood. We hypothesized that a correlation exists between plasma catecholamine metabolites and serum BDNF. In the current study, to examine this issue, we investigated correlations between serum BDNF and plasma levels of MHPG and HVA in the people with MD. The results might help shed light on catecholamine activities and BDNF in MD.

## Materials and Methods

### Participants

A total of 148 patients (male/female 65/83, age 49.5 ± 12.1 years old) were continuously recruited from 2004 to 2012 at Neuropsychiatry branch in University Hospital. Major depressive episodes were diagnosed using the Diagnostic and Statistical Manual of Mental Disorders IV Text Revision (15). The severity of depression was evaluated using the 17-item Hamilton Rating Scale for Depression (HAMD17) (16)-Japanese version. The evaluation was performed only once on the first day when the patients visited our branch in the hospital by Reiji Yoshimura (experienced psychiatrist over 30 years) according to the structural interview using GRID HAMD (17)-Japanese version. All patients had not taken any antidepressants at least 1 month before the evaluation. The people with MD patients whose HAMD17 scores 17 or more were enrolled in the present study. The exclusion criteria included any history of neurological disease or other physical diseases and comorbidities with other disorders, such as bipolar disorder or Axis II disorders based on the letters from former psychiatrists and/or family physicians. Blood samples and clinical evaluations were performed only once when patients first visited at our branch in the university hospital. The demographic data of the people with MD were described in Table 1. This study protocol was approved by the Ethics Committee of the University of Occupational and Environmental Health. Written informed consent was obtained from all subjects who participated in this study.

**Table 1:** Demographic data of the patients.

Number	148
M/F	65/83
Age (years old)	49.5 (12.1)
Episode (single/recurrent)	57/91
Melancholic/non-melancholic	47/101
Duration of illness (months)	2.3 (1.2)
Plasma methoxy-4-hydroxyphenylglycol (ng/mL)	5.9 (2.1)
Plasma homovanillic acid (ng/mL)	4.5 (1.5)
Serum brain-derived neurotrophic factor (ng/mL)	9.8 (2.9)

### Assays of Plasma Levels of HVA and MHPG

Blood was drawn at 9:00 a.m. Plasma levels of HVA and MHPG were analyzed in duplicate, and mean value were presented each data. Plasma HVA levels were analyzed using high-performance liquid chromatography with electrochemical detection (HPLC-ECD) in accordance with a slightly modified version of the method described by Yung et al. (18) In brief, each cyano-bonded solid-phase extraction cartridge was preconditioned with methanol, followed by glass-distilled water. The plasma sample (or the standard solution) and the internal standard solution (5-hydroxyindolecarboxylic acid) were added to each cartridge. The samples were then deproteinized with acetonitrile. The samples were mixed with vortex and centrifugation, then an aliquot of supernatant was allowed to very slowly pass through the cartridge under a mild vacuum. After the cartridge was washed twice with distilled water and extracted once with ethyl acetate, the aliquot was then evaporated to dryness under N_2_ and dissolved in a mobile phase (phosphate buffer). Finally, a 10-µL portion of the resulting solution was injected into the HPLC-ECD apparatus. The intra- and inter-assay coefficients of variation were 6.9 and 8.8%, respectively. The recovery rate was 83.1%.

Plasma MHPG levels were also analyzed using HPLC-ECD in accordance with a previously reported method (19). Briefly, after plasma was separated by centrifugation, extraction was performed under a vacuum using Bond-Elut columns (Varian, Palo Alto, CA, USA) prepacked with C_18_-bonded silica in a disposable syringe with a capacity of 1 mL. The columns, which were inserted into a vacuum chamber connected to an aspirator, were prepared by washing with 1 mL methanol followed by 1 mL of water. After the addition of an internal standard (vanillyl alcohol) to the plasma, the samples were very slowly passed through the columns, followed by two rinses with distilled water to wash off residual sample and easily eluted hydrophilic compounds. The adsorbed materials were eluted with methanol into a phosphate buffer mixture. Then, a 20-µL portion of this solution was then injected into the HPLC-ECD apparatus. The intra- and inter-assay coefficients of variation were 6.2 and 8.9%, respectively. The recovery rate was 81.0%.

### Assays of Serum BDNF

Blood was drawn at 9:00 a.m. Serum levels of BDNF were measured in duplicate using a Human BDNF ELISA Kit (Adipo Bioscience, Santa Clara, CA, USA), and mean value were presented each data. Protocols were performed in accordance with the manufacturer’s instructions mentioned as our previous study (20). Each well’s optical density was measured using an automated microplate reader (Emax; Molecular Devices, Sunnyvale, CA, USA).

### Statistical Analysis

Student’s *t*-test and Pearson’s correlation coefficient were used in two factors comparison. The Shapiro–Wilk test indicated that BDNF levels were normally distributed. A generalized linear model was used to explore BDNF level associated with HVA and MHPG levels, using potentially confounding variables (age, gender, first episode or not, melancholia or not, duration of untreated illness and HAMD17 scores at baseline) as covariates, and regarded the adjusted results as the study outcomes.

All statistical tests were performed using JMP (JMP 12, SAS Japan Inc., Tokyo, Japan), and *P* < 0.05 was considered to be indicative of statistical significance.

## Results

**Figure 1:**
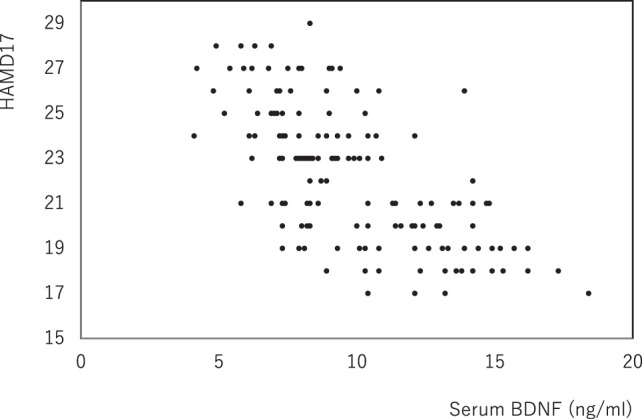
The changes in serum brain-derived neurotrophic factor (BDNF) and the HAMD17 scores. Data were analyzed by Pearson’s correlation coefficient (*r* = −0.654, *p* = 0.000).

The demographics of the people with MD enrolled were shown in Table 1. For the examined MDD patients, plasma HVA, plasma MHPG, and serum BDNF were 4.5 ± 1.5 (mean ± SD), 5.9 ± 2.1, and 9.8 ± 2.9 ng/mL, respectively (Table 1). When we performed non-adjusted simple two correlations of two factors among serum BDNF, plasma HVA, plasma MHPG, DUI, and the HAMD17, significant correlation was only found between serum BDNF and the HAMD17 (Figure 1). Serum BDNF levels in the people with MD with melancholia (8.6 ± 2.2 ng/mL) were significantly lower than in the people with MD with non-melancholia (10.3 ± 3.1 ng/mL). Serum BDNF, plasma HVA, and plasma MHPG were not different between in MDD patients with first episode, and those in recurrent (Table 2). No associations between plasma MHPG levels and age, sex, HAMD scores, or serum BDNF levels were identified (Table 3). In addition, no associations were detected between plasma HVA levels and age, sex, HAMD scores, or serum BDNF levels (Table 3). Serum BDNF levels were negatively associated with HAMD17 scores for the people with MD analyzed by (Table 3).

**Table 2:** Pearson’s correlations coefficient.

	Homovanillic acid (HVA)	Methoxy-4-hydroxyphenylglycol (MHPG)	Brain-derived neurotrophic factor (BDNF)	DUI	HAMD17T0
HVA (*r*)	1	−0.101	−0.4	0.158	0.011
(*p*)		0.221	0.626	0.055	0.897
MHPG	−0.101	1	0.049	−0.54	0.09
	0.221		0.558	0.514	0.914
BDNF	−0.4	0.049	1	−0.8	−0.654
	0.626	0.558		0.333	0
DUI	0.158	−0.54	−0.8	1	0.138
	0.055	0.514	0.333		0.093
HAMD17T0	0.11	0.09	−0.654	0.138	1
	0.897	0.914	0	0.093	

**Table 3:** Estimated value for each parameter revTKusing GLM model.

Factor	Estimate value	Standard error	Likelihood ratio ChiSq	*p*-Value (Prob > ChiSq)	Lower trust limit	Upper trust limit
Intercept	24.60465	1.938295	109.0126	<0.0001	20.78088	28.42842
Age	−0.01156	0.015162	0.579953	0.4463	−0.04147	0.018353
Gender	0.416807	0.373747	1.2385	0.2658	−0.3205	1.154117
Methoxy-4-hydroxyphenylglycol	0.06236	0.084592	0.542453	0.4614	−0.10452	0.229239
Homovanillic acid	−0.07711	0.121795	0.400246	0.527	−0.31738	0.163165
HAMD17T0	−0.67661	0.06799	75.82338	<0.0001	−0.81074	−0.54249
Status[reurence]	0.389329	0.206215	3.522217	0.0606	−0.01748	0.79614
Duration of illness (m)	0.02451	0.144857	0.028626	0.8656	−0.26126	0.310276
Mel[melancholia]	0.061025	0.219801	0.077061	0.7813	−0.37259	0.494638

## Discussion

The main finding of the present study was that plasma levels of catecholamine metabolites were not associated with serum BDNF levels, even though catecholamine might influence BDNF synthesis and secretion in the brain (21) and BDNF might play an important role in the synthesis and/or secretion of catecholamine (22). We have previously reported a negative correlation between plasma MHPG and serum BDNF in healthy workers (13, 14). In this study, no correlation between serum BDNF and plasma MHPG or HVA was observed in the people with MD. Given the aforementioned findings, it is plausible that individuals’ sympathetic nerves are activated by stress, with potential imbalances when the people with major depressive state is reached. Most BDNF in serum is derived from platelets because platelets are abundant in serum. However, plasma BDNF might be derived from the brain because there are few platelets in plasma. We reported that serum BDNF levels were 14-fold higher than plasma BDNF levels, and a close relationship was found between serum and plasma BDNF levels (23). Pan et al. demonstrated that intact BDNF in the peripheral circulation crosses the BBB by a high-capacity, saturable transport system (24). The time course of central and peripheral BDNF changes might differ, it can be, however, justified to measure serum BDNF levels with a time delay to monitor brain tissue neurotrophin alterations in rats (25). From taking these findings into account, blood (serum or plasma) partially reflect the brain.

A recent meta-analysis indicated that serum levels of mature BDNF are lower in patients with MDD than in healthy controls. Serum levels of mature BDNF were found to be initially low but to increase over the course of antidepressant treatment (26). Although there was no control group in the present study, we could not compare in the MDD group and healthy subjects regarding plasma level of catecholamine metabolites or serum BDNF. Plasma HVA, plasma MHPG, and serum BDNF in healthy subjects from our pooled data in our lab (*n* = 218, male/female 122/96, age 34.7 ± 14.2 years old) were 5.2 ± 2.3 (mean ± SD), 4.3 ± 1.9, and 12.1 ± 3.6 ng/mL, respectively. However, we could not compare above data, because the experiments were performed different time, age and genders were not matched, and also factors influence BDNF, MHPG, HVA were heterogeneous in the both group. For example, body mass index (27), smoking (28), and personality traits also influence serum BDNF levels (29). In short, the two groups have different back ground, respectively. We have previously reported a significant correlation between HAMD17 scores and serum BDNF levels (17). In the present study, we reconfirmed this prior finding in a more robust manner. The results indicate that serum BDNF is a useful candidate biomarker for depressive severity in MDD patients. Serum BDNF was not correlated with plasma MHPG or plasma HVA in the people with MD. The results of the present study suggest that BDNF and catecholamine metabolites are not related in the periphery despite the fact that catecholaminergic neurons have important correlations with BDNF in the brain. Psychological stress generally enhances catecholaminergic activity, although there may be individual differences with respect to this reaction. One group of the people with MD might have high levels of MHPG or HVA, whereas another group of the people with MD might have low levels of MHPG or HVA; this difference may be partially determined by genetic factors such as polymorphisms. Interestingly, it has been reported that the regulation of serotonin influences both serotonin metabolites and catecholamine metabolites (30). 5-HTT is believed to affect the pathogenesis of mood disorders. Numerous genetic association studies have examined how 5-HTT functional polymorphisms relate to vulnerability to mood disorders and therapeutic responses to antidepressants. We have investigated the effects of the 5-HTT-linked polymorphic region (5-HTTLPR) and concentrations of monoamine metabolites in CSF for treatment-resistant patients with mood disorders and found a higher HVA concentration in S/S subjects than in L/L and L/S subjects and significantly higher hydroxyindole acetic acid (5-HIAA) concentrations among carriers of an S allele than among other individuals (31). Yoon et al. (7) reported that all monoamine metabolites (MHPG, HVA, and 5-HIAA) were significantly lower in moderately to severely depressed patients than in controls; they also found that antidepressants decreased levels of 5-HIAA and MHPG. In contrast, antidepressants increased HVA levels, suggesting that HVA might be useful in clinical settings (7).

Luykx et al. (32) conducted a genome-wide association study of monoamine metabolite levels in CSF for 414 human subjects from the general population. In a linear model with corrections for covariates, the authors identified one locus associated with monoamine metabolites that exhibited genome-wide significance; this locus was 20 kb from SSTR1, a gene involved in brain signal transduction and glutamate receptor signaling. Those authors performed a subsequent quantitative trait locus analysis for whole-genome expression that provided evidence that this variant controls the expression of PDE9A, a gene previously implicated in monoaminergic transmission, MD and antidepressant response; these findings will hopefully contribute to an exploration of the functional impact of the highlighted genes on monoaminergic transmission and neuropsychiatric phenotypes (32).

Based on the aforementioned findings, the transmission of catecholamine appears to be complicated and to involve many interactions. Levels of catecholamine metabolites are regulated by many genes. With respect to BDNF, we have previously reported that the BDNF Val66Met polymorphism is not correlated with serum BDNF levels (11).

## Conclusion

Our hypothesis was not supported. In short, there was no strict and direct correlation between catecholamine metabolites (MHPG and HVA) and BDNF in the blood for the people with MD. The implication of this study is that plasma levels of catecholamine metabolites neither influence nor are influenced by serum BDNF levels. The present preliminary study had several serious limitations, such as the absence of a control group, a small sample, heterogeneous, and cross-sectional design. Thus, we are performing research longitudinal study with a large sample, with age- and sex-matched control group to reconfirm these preliminary results.

## Highlight of the Article

- No correlation was found between plasma catecholamine metabolites (MHPG and HVA) and serum BDNF for the people with MD.
- A robust correlation was found between serum BDNF and the HAMD17.

## Ethics Statement

All subjects were provided with information about the procedures. Written informed consent was obtained via forms approved by the local ethics committee of the University of Occupational and Environmental Health.

## Author Contributions

RY, TK, KA, and AK conceived and designed the experiments, corrected blood samples, evaluated HAMD17 scores, measured plasma catecholamine metabolites and serum BDNF levels, analyzed the data, and wrote the first draft of the manuscript. NI read and corrected this first draft and approved the final manuscript.

## Conflict of Interest Statement

RY, TK, KA, AK, and NI declare that they have no direct conflicts of interest relevant to this study. No grant support or other sources of funding were used to conduct this study or prepare this manuscript. RY has received speaker’s honoraria from Daiichi Sankyo, Dainippon Sumitomo, Eisai, Janssen, Otsuka, Meiji, MSD, and Eli Lilly. TK has received speaker’s honoraria from Daiichi Sankyo, Dainippon Sumitomo, Eisai, Janssen, Otsuka, Meiji, MSD, and Tanabe-Mitsubishi (Yoshitomi) and has received a Health Labour Sciences Research Grant and a Fujita Health University School of Medicine research grant. NI has received speaker’s honoraria from Astellas, Dainippon Sumitomo, Eli Lilly, GlaxoSmithKline, Janssen, Yoshitomi, Otsuka, Meiji, Shionogi, Novartis, and Pfizer and has received research grants from GlaxoSmithKline, Meiji, and Otsuka.
